# Multiple time scales of the ventriloquism aftereffect

**DOI:** 10.1371/journal.pone.0200930

**Published:** 2018-08-01

**Authors:** Adam K. Bosen, Justin T. Fleming, Paul D. Allen, William E. O’Neill, Gary D. Paige

**Affiliations:** 1 Department of Biomedical Engineering, University of Rochester, Rochester, NY, United States of America; 2 Department of Neurobiology and Anatomy, University of Rochester, Rochester, NY, United States of America; University of Muenster, GERMANY

## Abstract

The ventriloquism aftereffect (VAE) refers to a shift in auditory spatial perception following exposure to a spatial disparity between auditory and visual stimuli. The VAE has been previously measured on two distinct time scales. Hundreds or thousands of exposures to a an audio-visual spatial disparity produces enduring VAE that persists after exposure ceases. Exposure to a single audio-visual spatial disparity produces immediate VAE that decays over seconds. To determine if these phenomena are two extremes of a continuum or represent distinct processes, we conducted an experiment with normal hearing listeners that measured VAE in response to a repeated, constant audio-visual disparity sequence, both immediately after exposure to each audio-visual disparity and after the end of the sequence. In each experimental session, subjects were exposed to sequences of auditory and visual targets that were constantly offset by +8° or −8° in azimuth from one another, then localized auditory targets presented in isolation following each sequence. Eye position was controlled throughout the experiment, to avoid the effects of gaze on auditory localization. In contrast to other studies that did not control eye position, we found both a large shift in auditory perception that decayed rapidly after each AV disparity exposure, along with a gradual shift in auditory perception that grew over time and persisted after exposure to the AV disparity ceased. We modeled the temporal and spatial properties of the measured auditory shifts using grey box nonlinear system identification, and found that two models could explain the data equally well. In the power model, the temporal decay of the ventriloquism aftereffect was modeled with a power law relationship. This causes an initial rapid drop in auditory shift, followed by a long tail which accumulates with repeated exposure to audio-visual disparity. In the double exponential model, two separate processes were required to explain the data, one which accumulated and decayed exponentially and the other which slowly integrated over time. Both models fit the data best when the spatial spread of the ventriloquism aftereffect was limited to a window around the location of the audio-visual disparity. We directly compare the predictions made by each model, and suggest additional measurements that could help distinguish which model best describes the mechanisms underlying the VAE.

## Introduction

The ability to associate auditory and visual spatial information requires the nervous system to maintain spatial congruence between two distinct spatial encoding mechanisms. The visual system directly encodes location by retinotopic mapping and binocular disparity, whereas the auditory system computes location from binaural time and level differences, and monaural spectral cues [1–3]. Congruence must be maintained despite changes in cue mapping to the external world, which can occur as a result of development, disease, injury, or therapeutic modifications (e.g. corrective eyeglasses) [4]. When spatial incongruence occurs between audition and vision, auditory perception adjusts to match vision [5–10], because perception is weighted toward the typically more precise spatial information provided by vision [11–13]. Shifts in auditory spatial perception in response to audio-visual (AV) spatial disparities are generally referred to as the ventriloquism aftereffect (VAE).

Previous studies of the ventriloquism aftereffect have often focused on a single time scale. Early studies of the ventriloquism aftereffect measured auditory spatial perception before and after repeated exposure to a constant AV spatial disparitiy [6, 7], and found that encoded auditory location shifted to compensate for the offset. More recent studies have shown that repeated exposure is not necessary, but rather that shifts in auditory spatial perception can be observed after as little as a single exposure to an AV spatial disparity [14]. These studies indicate that the ventriloquism aftereffect can be observed on multiple time scales, but do not demonstrate how these observations are related to one another. Some studies have attempted to measure the time course of the ventriloquism aftereffect but have failed to control for such as eye position, which can produce confounding shifts in auditory spatial perception [15]. The goal of the current study was to measure the time course of auditory shift in response to extensive exposure to AV spatial disparity, while ensuring that shifts could not be attributed to eye position rather than the ventriloquism aftereffect.

Wozny and Shams [14] first demonstrated that exposure to a single AV spatial disparity could produce measurable shifts in encoded location of subsequent auditory targets. This shift occurred in auditory targets following an AV spatial disparity regardless of where the auditory target was presented, and gradually accumulated with repetition of AV spatial disparities in the same direction. In our previous work [16], we extended these findings by measuring how shifts in auditory spatial perception decay over time and diminish with distance from the location of the AV disparity. Specifically, we found that shifts were most prominent 1 s after exposure to an AV disparity, and that by 20 s the auditory shift had decayed to the point where it was not significantly different from zero. However, at the 20 s time point the shift still trended in the same direction as the initial shift, so it is possible that this small residual shift forms the basis for slower, more persistent forms of adaption. Auditory targets that were ±15° from the auditory component of the AV disparity also showed lower shift than auditory targets presented at the same location as the auditory component of the AV disparity, although they were still significantly greater than zero. This spatial effect is in agreement with previous studies that have shown that the ventriloquism aftereffect is specific to the location of the AV disparity [10, 17]. We also found that auditory shift was greater for 20 repetitions than for one repetition of the AV disparity. This finding suggests that auditory shift accumulates with repeated exposure, in agreement with [14]. The presence of both accumulation and decay produced by brief bursts of AV spatial disparities suggests that the time course of auditory spatial shifts is unlikely to be monotonic with repeated exposure to a fixed AV spatial disparity.

When individuals are exposed to a fixed AV spatial disparity for an extensive period of time (hundreds to thousands of expsoures to a consistent disparity), their perception of auditory space shifts to compensate for the disparity [7, 8, 18, 19]. In contrast to the shift produced by brief disparities, this shift produced by extensive exposure does not decay over time, at least within the time period over which localization is measured following exposure. Shifts in response to extensive exposure are also specific to the frequency of the auditory target used during exposure [7], in constrast to shifts produced by brief disparities [20]. These findings suggest that shifts produced by brief disparity may be distinct from shifts produced by extensive exposure. Therefore, we expect to find evidence that auditory shifts in response to brief disparities occur in conjunction with slower shifts in response to extensive exposure.

Some previous studies have attempted to study the time course of auditory shift in response to repeated exposure, but have either introduced a manipulation that could alter the time course [20], or did not control for eye position during exposure [21–24]. When individuals maintain an eccentric eye postion relative to the head, auditory spatial perception shifts in the direction of gaze [13, 15, 25]. As a result, if visual target locations used during repeated exposure to an AV spatial disparity are not distributed uniformly around straight-ahead and individuals are allowed to look at visual targets, then eye position will be eccentric relative to the head on average. This eccentric eye position will produce auditory shifts, in addition to the AV spatial disparity. In one previous study that did not control for eye position, the reported time course and magnitude of the auditory shift observed in response to an AV spatial disparity [23] was similar to the time course and magnitude of the eye position effect previously reported in [15], suggesting that their data measured the combination of both effects, rather than just the effect of the AV spatial disparity as they intended.

To measure the evolution of shifts in auditory spatial shifts in response to AV spatial disparities over multiple time scales, subjects localized auditory targets before, after, and during extensive repetition of a fixed AV disparity presented at several azimuths. Differences in localization before and after exposure to the fixed AV disparity quantified long term shifts in auditory spatial perception. Localization of isolated auditory targets interleaved with the fixed AV disparity measured auditory shift following each AV spatial disparity, and allowed us to observe the gradual buildup of long term shifts in auditory spatial perception with repeated exposure to disparity. Our findings demonstrate that transient effects of visual capture on auditory spatial perception are superimposed on persistent shifts in auditory spatial perception. We then used this data to identify which models of auditory shift in response to AV spatial disparity best explain the data. Our results indicate that models that included either two exponential temporal processes or a single power law decay process could fit the data equally well.

## Experimental methods

### Subjects

Twelve volunteers (5 male; age 19-27) recruited from the Rochester, New York community participated in this experiment. All subjects were screened for normal hearing (thresholds less than 20 dB HL, at octave frequencies from 250 Hz to 8 kHz) and normal or corrected-to-normal vision.

Experimental protocols were approved by the Institutional Review Board at the University of Rochester and were performed in accordance with the 1964 Declaration of Helsinki. All subjects provided written informed consent and were compensated at a rate of $10 per hour for their participation.

### Apparatus

Experiments were conducted in a dark, sound-attenuated chamber designed for the presentation of auditory and visual targets from a range of locations in front of subjects (for additional detail, see [16]). Subjects were seated 2 meters from a speaker, which was mounted on a robotic arm. The arm was hidden from sight by a black, acoustically transparent speaker cloth, which was mounted on a cylindrical frame approximately 1.9 meters from the subject and subtended ±90° in azimuth and extended approximately ±20° in elevation. When positioning the speaker, sound produced by the robotic arm was masked by continuous Gaussian white noise presented at 65 dB SPL from two speakers behind the speaker cloth and outside the spatial range of targets used in this experiment (±75° azimuth, +20° elevation). The motor that rotated the arm was located under the subject, so sound made by the motor was uninformative as to speaker position. Subjects could tell that the movement was occurring, but were unable to identify the direction or magnitude of movements. Additionally, timing cues associated with the arm movement duration were decoupled from movement distance by moving the speaker to a random azimuth, then to the target location, every time the speaker moved. This made the inter-trial interval independent of how far the target moved between successive trials.

Subjects were head-fixed via bite bar, which was oriented for each subject such that Reid’s baseline was horizontal and the point midway between their eyes was pointed at the origin of the room (0° azimuth). Eye movements were continuously monitored by electrooculography. A LED pointer was mounted on a cylindrical drum directly below the bite bar, which subjects could rotate in pitch and yaw to indicate auditory target location. Eye position, pointer and target locations, and stimulus onset and response times were sampled at a rate of 1 kHz by a real-time LabVIEW system (National Instruments, Austin, TX). Data analysis was performed in MATLAB (MathWorks Inc., Natick, MA).

### Stimuli

Auditory targets were 50 ms broadband frozen noise bursts, which had a flat energy spectrum between 0.2–20 kHz, were presented at 65 dB SPL, and were gated on and off with 1 ms *cos*^2^ ramps. All auditory stimuli were generated by a TDT RX8 Multi I/O Processor (Tucker-Davis Technologies, Alachua, FL) and played from the speaker mounted on the robotic arm. Visual stimuli (brightness 5 cd/m^2^, 0.1° subtended angle) were projected onto the speaker cloth from a green laser projecting into an X-Y mirror-galvanometer mounted over the subject’s head. A red laser, which projected onto the speaker cloth at 0° azimuth and elevation, was illuminated between trials to provide a fixation reference.

### Experimental procedure

**Fig 1:**
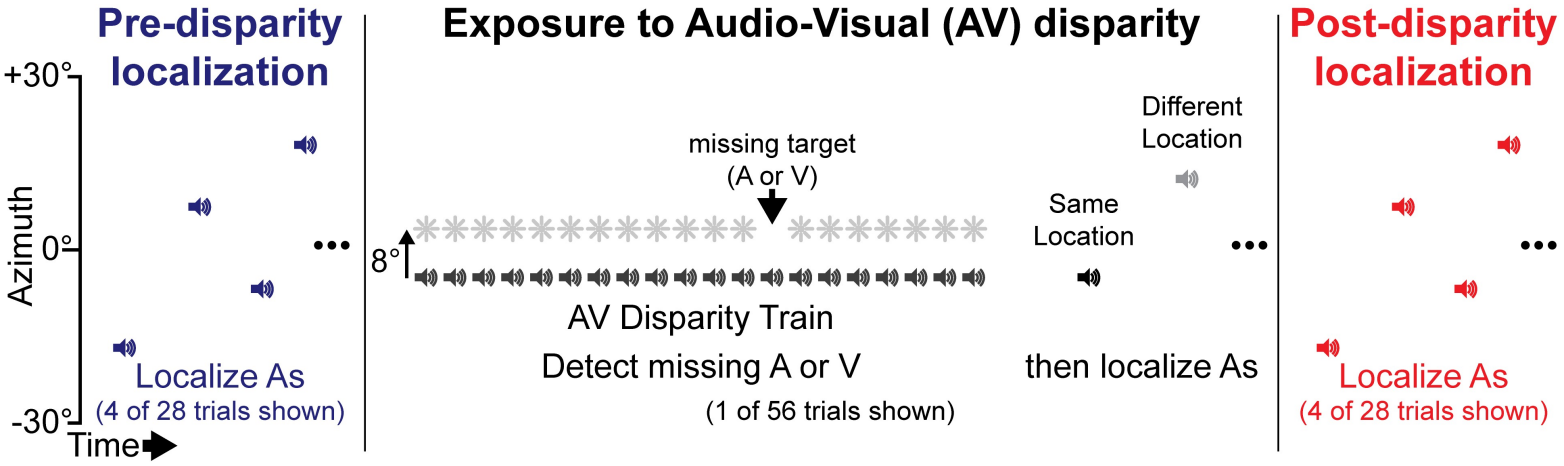
Experimental timeline. In each experimental session, subjects localized auditory targets (represented by speaker symbols) at a range of azimuths before and after repeated exposure to an audio-visual spatial disparity, to observe long-term shifts in auditory perception produced by exposure. Subjects also localized auditory targets following each AV disparity, to observe the effect of each disparity on auditory perception.

Each subject performed two experimental sessions on different days within two weeks of one another, depending on the subject’s availability. Each session comprised three blocks of trials (Fig 1). First, in the *pre-disparity localization* block, subjects localized auditory targets in isolation to establish a localization baseline. Second, in the *exposure to AV disparity* block, subjects localized the same set of auditory targets between repeated exposures to a fixed leftward or rightward AV disparity, to measure the time course of shifts in auditory localization caused by exposure to the AV disparity. Third, in the *post-disparity localization* block, subjects localized the same set of targets as in the pre-disparity localization block, to measure any enduring changes in localization that might persist after exposure to the AV disparity.

In the pre- and post-disparity localization blocks, auditory targets were presented in isolation at azimuths ranging from −30° to +30° in 3° increments (excluding 0°), with targets at ±6° and ±15° presented three times each and all other locations presented once (a total of 28 localization trials in each block). The LED pointer was illuminated 200 ms after the end of each target, signaling that the subject should look and guide the pointer to the remembered target location, then press a button to register their response. The same sequence of targets was used in the pre- and post-disparity localization blocks, to allow for paired comparison of any changes that resulted from the exposure to AV disparity block.

In each exposure to AV disparity trial, subjects viewed an AV disparity train, then localized one, two, or four auditory targets presented in isolation after the end of the AV disparity train. For each session, AV disparity was fixed so that the visual target was always either leftward (−8° azimuth) or rightward (+8° azimuth) of the auditory target (order counterbalanced across subjects). All AV disparities were presented with the auditory target at a pseudorandom location between −30° to +30° in 3° increments (excluding 0°), to match the range of locations used in the pre- and post-disparity blocks. Visual targets were offset relative to the auditory targets, so they presented within a range of −38° to +22° for leftward disparities and −22° to +38° for rightward disparities.

Each AV disparity train contained 20 repetitions of a pair of AV targets presented synchronously at 4 Hz that were separated by 8° in azimuth. Previous studies that showed an enduring recalibration of auditory space used hundreds to thousands of exposures to an AV disparity [7, 8, 19], so to increase the total number of exposures we used 20 repetitions for each trial. This also has the effect of increasing the magnitude of the auditory shift following each AV disparity train, because 20 repetitions produces a greater shift than a single repetition [16]. Each repetition was 50 ms long, producing a temporal sequence for each AV disparity train of 50 ms on, 200 ms off, repeated for 5 seconds. In order to maintain attention to both modalities during AV disparity trains, one target was dropped from either the visual or auditory stream, and subjects were instructed to press a button as quickly as possible whenever they detected an unpaired target. Unpaired targets occurred with equal frequency in each modality, between the 3rd and the 19th target in the AV stimulus train. Reaction times and false alarm rates were quantified to assess attentiveness during exposure trials. Detection of unpaired targets was at or near 100% for all subjects and false alarm (responses before or more than 1 second after the unpaired target) rates were at or near 0%, indicating that subjects sustained attention throughout the experiment.

To observe the time course of auditory spatial shift during the exposure to AV disparity block, each AV disparity train was followed by one, two or four auditory targets presented and localized in isolation. Following each auditory target, the LED pointer was illuminated and subjects localized the target as in the pre and post-disparity localization blocks. For each AV disparity train, an isolated auditory target was presented approximately 1–3 seconds after the end of the AV disparity train. This auditory target was always presented at the same location as the auditory target in the preceding AV disparity train, to avoid any spatial influence on auditory shift from that disparity train. These auditory targets are referred to as “same location” trials in the following data analysis. The time delay before isolated auditory targets was variable across trials due to issues with the robotic arm control software at the time of testing, and was random from trial to trial and across subjects. On 32 trials, a second auditory target was presented following a 8–12 s delay from another location, pseudorandomly selected from between −30° to +30° azimuth in 3° increments (excluding 0°). On 8 of these trials, we also presented a third and fourth target under the same conditions as the second target, to ensure that we were sampling the ventriloquism aftereffect at a variety of times. Collectively, the second, third, and fourth targets are referred to as “different location” trials in the following analysis. These trials were grouped in the experimental analysis because time and distance from the preceding AV disparity had poor ability to predict auditory shift across the group (see S1 File for fit details). Detailed analysis of the spatial and temporal properties of the ventriloquism aftereffect was reserved for the modeling section. We did not include different location trials following every AV disparity train to keep the overall experiment time tolerable to our subjects.

In total, subjects viewed a total of 1064 repetitions of the AV disparity (in trains of 20, less one unpaired target from the attention task) over 56 AV disparity trains. Subjects were given a 5 minute break in darkness halfway through the exposure to AV disparity block to minimize fatigue. An experimental session took between 43–49 minutes total, depending on the speed at which subjects localized targets.

Because eye position biases unimodal auditory spatial perception [13, 15, 25], subjects maintained center fixation except when localizing auditory targets. The fixation reference was illuminated between trials and was extinguished 100 ms prior to the onset of a trial. Subjects were instructed to maintain center fixation during target presentation without this visual reference. Subjects were allowed to move their eyes to guide the LED pointer when localizing, because eye movements after target presentation do not bias localization of remembered targets [13]. Eye position was continuously monitored by the experimenter to detect any breaks in fixation during target presentation, but none were observed.

The LED pointer was on between trials and when localizing remembered targets, but off during target presentation. Between trials, subjects pointed the LED pointer to the fixation reference and held this position throughout stimulus presentation, to ensure that each localization response started from the same location.

## Experimental results

**Fig 2:**
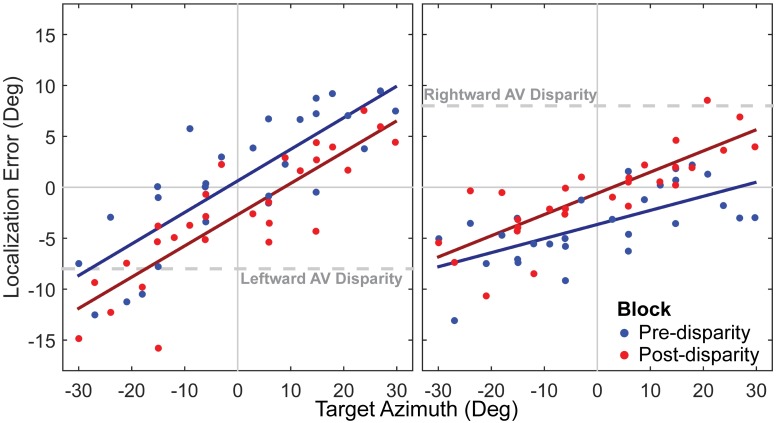
Example pre- and post-disparity auditory localization. **left panel**, Localization responses from one subject before and after exposure to leftward (visual −8° azimuth relative to auditory) AV disparity. Blue and red circles represent localization response error as a function of target location in the pre-disparity and post-disparity blocks, respectively, and lines of the same color represent a linear fit to the data from each block. **right panel**, Shift in the same subject from exposure to rightward (visual +8° azimuth relative to auditory) disparity.

**Fig 3:**
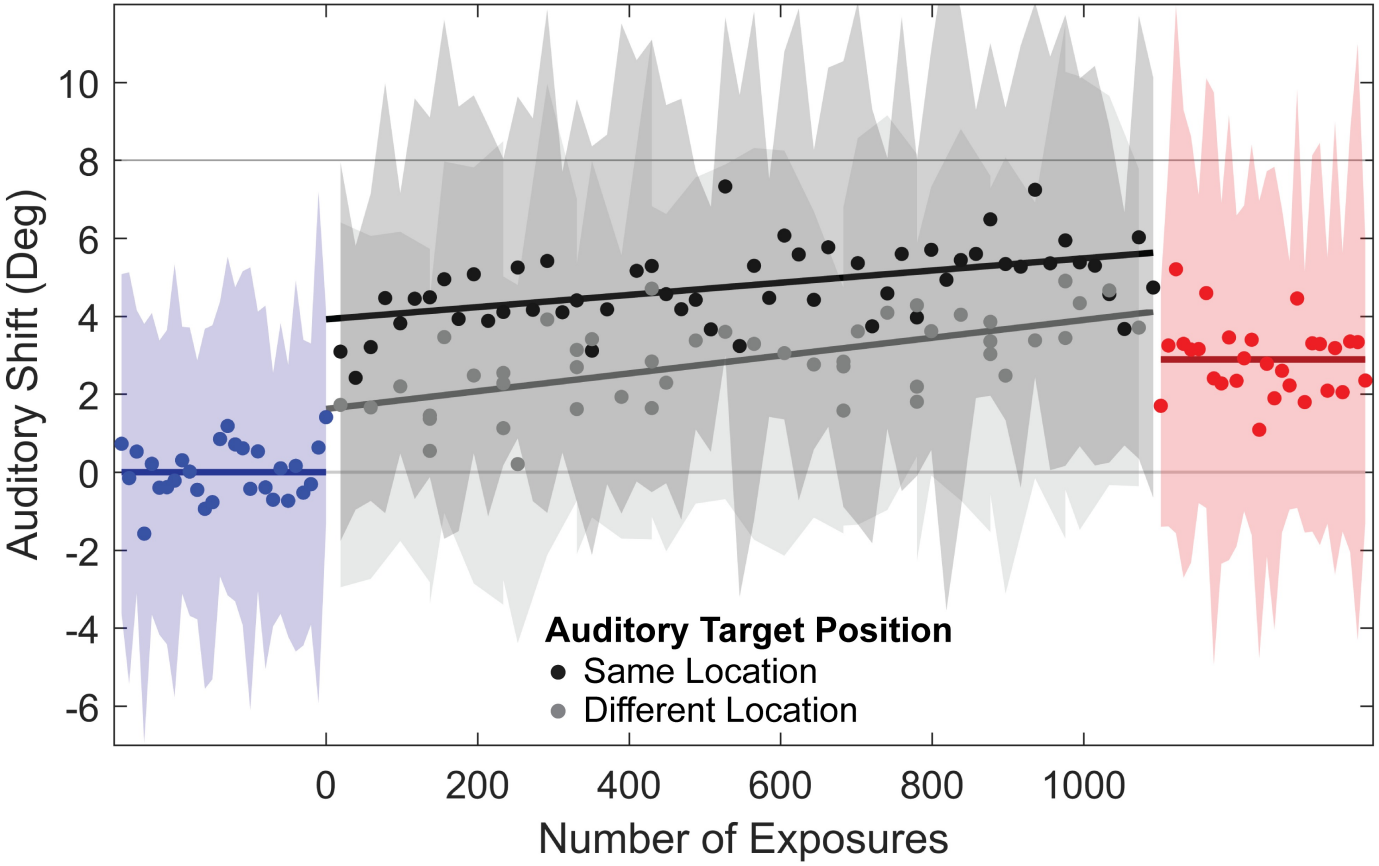
Evolution of auditory shift with repeated exposure to fixed AV disparity, averaged across subjects. Blue and red points represent average relative error for pre-disparity and post-disparity, respectively. Black and gray points represent average relative error while localizing auditory targets during the exposure block, with black indicating localization of auditory targets presented 1–3 seconds after and 0° away from the auditory component of the AV disparity (“same location” targets), and gray indicating localization of targets presented more than 8 seconds after and at a different location than the auditory component of the AV disparity (“different location” targets). Blue and red lines indicate mean average relative error in the pre and post disparity blocks (with auditory shift defined relative to the pre-disparity localization responses for each session), gray lines show linear best fits to responses in the exposure block, and shaded regions represent ±1 standard deviation across subjects for each sample of the corresponding color.

**Fig 4:**
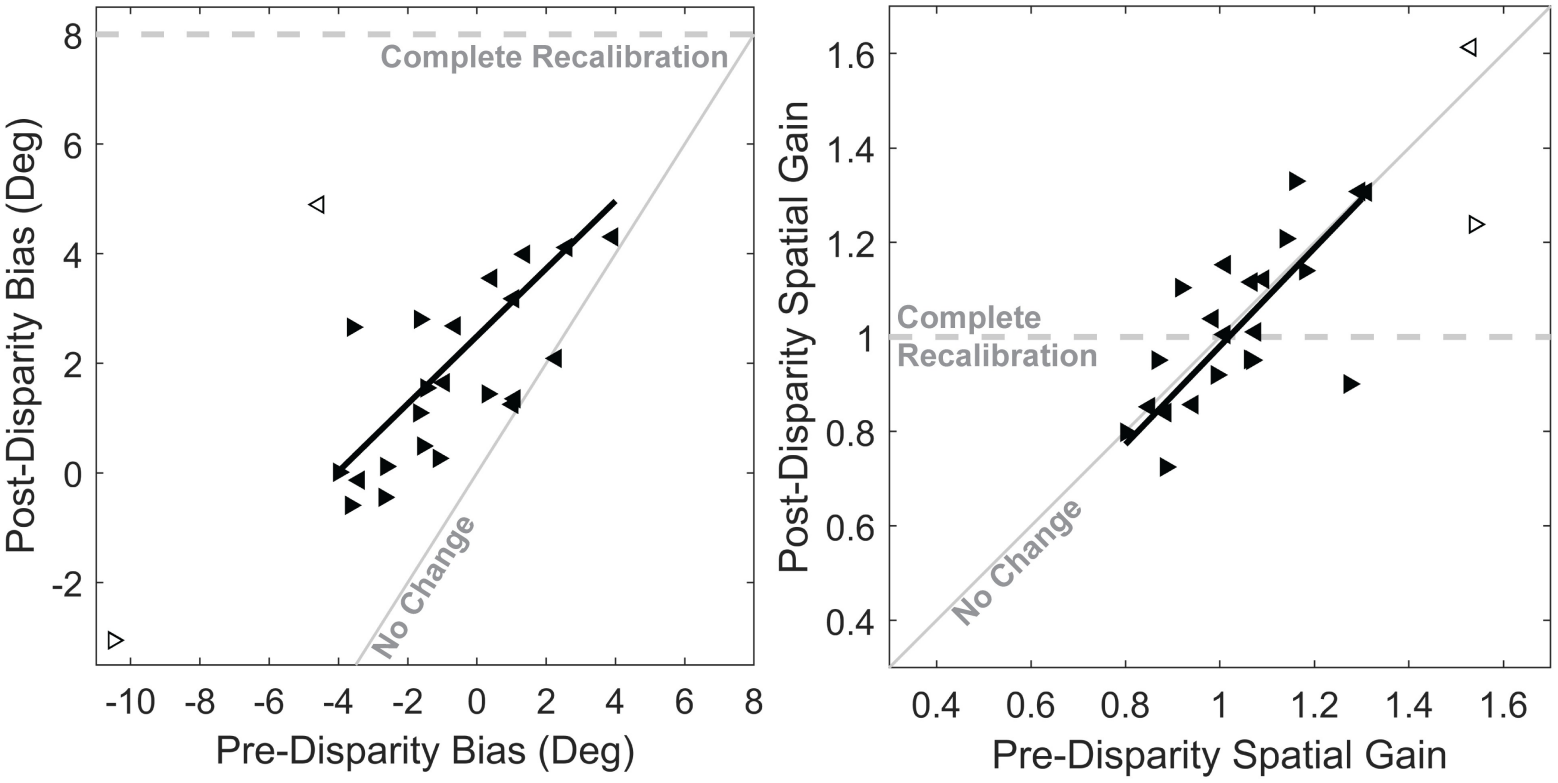
Pre- and post-disparity comparison of auditory bias and gain. **left panel**, Difference in bias between the pre-disparity and post-disparity blocks. Data from leftward and rightward disparity sessions are represented with leftward and rightward pointing arrows, respectively. The two hollow symbols represent one subject that was an apparent outlier, and was excluded from the line fit. Data from leftward disparity sessions was mirrored through the origin to overlay rightward data. The dashed gray line represents where responses would fall if subjects completely compensated for encoded disparity, the solid gray line indicates where responses would fall if no change in bias was observed, and the solid dark line is a linear best fit. **right panel**, Difference in spatial gain between the pre-disparity and post-disparity blocks.

An example of the shift in auditory perception caused by repeated exposure to a fixed AV disparity is shown in Fig 2. To determine the time course of shifts in auditory spatial perception in response to repeated exposure to a fixed AV disparity, we averaged localization responses across listeners as a function of time, as shown in Fig 3. Next, we performed a within-subject comparison of pre-disparity and post-disparity differences in auditory localization, to determine the magnitude of auditory spatial recalibration and the influence of individual variability on recalibration, as shown in Fig 4.

### Changes in auditory spatial perception following repeated exposure to fixed AV disparity

Fig 2 shows changes in auditory localization from the pre-disparity to the post-disparity block in one example subject for both leftward and rightward shift experimental sessions. As shown, responses in the post-disparity block are shifted in the direction of the disparity in both experimental sessions, indicating that exposure to the AV disparity elicited a compensatory shift in auditory spatial perception. Across all listeners, responses tended to show a positive slope (bias toward the periphery) and small offsets in the pre-disparity block. To account for these initial inaccuracies in auditory perception, a linear fit was applied to each listener’s responses in the pre-disparity block as a function of target location, and *auditory shift* was calculated as the difference between all localization responses and the linear fit for each experimental session. This definition of auditory shift allowed us to compare localization responses over time in the exposure and post-disparity blocks to responses in the pre-disparity block. Additionally, the sequence and location of auditory targets were identical in the pre-disparity and post-disparity blocks, which allowed for paired comparisons of localization responses across each target location. Across the group, paired differences in responses between the pre-disparity and post-disparity blocks ranged from 0° to 8°, with a mean of 2.9°. A one-way t-test indicated that the mean difference was significantly greater than zero (*t*(23) = 6.23, *p* = 1.2 × 10^−6^).

#### Temporal dynamics of shifts in auditory spatial perception

Fig 3 shows the shift in auditory localization throughout an experimental session. Results were normalized across listeners so that positive auditory shift was toward the visual component of the AV disparity, and then averaged without binning or smoothing for each trial across all subjects and sessions. As a result, auditory shift has zero mean in the pre-disparity block, and positive values of auditory shift in the exposure and post-disparity blocks indicate that auditory perception moved from the intial encoded auditory target location toward the visual target.

Responses during the exposure to AV disparity block were split into two groups: auditory targets presented 1–3 seconds after exposure to AV disparity at the same location as the auditory component of the AV disparity were referred to as “same location” targets, and targets presented more than 8 seconds later and at a different location than the auditory component of the AV disparity were referred to as “different location” targets. Based on our previous study of the time course of auditory shift following an AV disparity train [16], we expected that the time delay and spatial separation would reduced the auditory shift produced by the preceding AV disparity train to nearly zero for all different location targets, so we averaged them together in this analysis.

Simple linear regression of the same location and different location responses against the number of exposures revealed a significant relationship. Linear fits had *R*^2^ values of 0.24 (F(2,54) = 18.4, *p* < 0.001) for the same location responses and 0.38 (F(2,46) = 29.9, *p* < 0.001) for the different location responses. Slopes for both fits were significant (*p* < 0.001 for both fits), indicating a gradual recalibration of auditory localization over time. The intercepts of these fits were significantly greater than zero (*p* < 0.001 for both fits), indicating that auditory shift immediately appears after a single exposure. This was surprising for the different location responses, because our previous work [16] indicated that the ventriloquism aftereffect should be nearly zero at those distances and delays from the preceding AV disparity train. A Welch two sample t-test demonstrated that auditory shift is significantly larger for same location targets than for different location targets (uneven number of samples, *t*(102) = 9.24, *p* < 0.001), indicating that auditory shift decays over time and/or with distance from AV exposure location. Simple linear correlations indicated that auditory shift was uncorrelated with time in the post-disparity block across the group (*p* = 0.43). This suggests that auditory shift persists for at least as long as the time required to measure localization in the post-disparity block.

#### Spatial patterns of shifts in auditory spatial perception

As shown in Fig 2, localization responses are subject to systematic inaccuracies (perfect performance would be a flat line with zero intercept). Specifically, localization responses are subject to both uniform errors across space (referred to as *bias*, *μ*_*A*_) and a tendancy to overestimate target eccentricity (referred to as positive *spatial gain*, *SG*) [9, 15, 26, 27]. As a consequence, the disparity between the auditory and visual locations *encoded* by the subject’s sensory systems can differ from the *physical* disparity, as shown in Eq 1. We specifically refer to this difference as encoded disparity to avoid confusing it with the holistic percept produced by the AV disparity, because we did not measure perceived visual location and therefore do not know how subjects perceived the AV stimulus as a whole.

$$\begin{array}{c} \Delta_{\text{Encoded}}=S_{V}-\left(SG*S_{A}+\mu_{A}\right) \end{array}$$ (1)

*S*_*V*_ and *S*_*A*_ represent visual and auditory target locations, respectively. Visual localization errors are assumed to be negligible.

If we assume that *encoded* AV disparity, Δ_Encoded_, drives shifts in auditory perception, then recalibration should be sensitive to bias and spatial gain. Since bias is uniform across space, it has the net effect of producing a mean encoded disparity that is the sum of physical disparity (8°) and bias. For example, if a subject’s initial bias is equal to the AV disparity (both are 8°), the mean encoded AV disparity would be zero, and there would be no need to shift auditory spatial perception. However, if initial bias differs from the physical AV disparity, encoded disparity would grow in proportion to the difference between the physical disparity and initial bias. The left panel of Fig 4 shows the relationship between pre and post-disparity bias. As shown, the trend across subjects (dark line, model II Standard Major Axis Regression [28]) has a slope that is shallower than the “no change” (95% confidence interval for slope ranges from 0.4 to 0.83). This indicates that the difference in bias between the pre and post-disparity blocks is a function of the pre-disparity bias, confirming that auditory shift is sensitive to mean encoded AV disparity. On average, responses in the pre-disparity block showed a small but significant leftward bias of −1.6° across all experimental sessions (which is negative for the rightward arrows and positive for the leftward arrows plotted in Fig 4), as confirmed with a one-sample t-test (*t*(23) = −2.78, *p* = 0.01). A small leftward bias has been previously observed in our lab [27] and in other labs [29]. As a result, the distribution of pre-disparity bias is segregated by the direction of the session in Fig 4, which subsequently makes Δ_*Encoded*_ uneven across leftward and rightward sessions. Therefore, it is possible that the trend shown could alternatively be explained by different rates of auditory shift across the leftward and rightward directions, although we are not aware of any previous findings that would support this alternative explanation.

In contrast, Fig 4 demonstrates that there is no significant change in spatial gain in this experiment. Pre-disparity spatial gain reflects the proportional change in localization error as a function of target location, *S*_*A*_. If subjects were sensitive to the pattern of disparity across space, they should demonstrate greater auditory shift in regions of space where encoded disparity is large. In other words, changes in *SG* should act to correct for any undershoot or overshoot (*SG* ≠ 1), as represented by the dashed gray line in the right panel of Fig 4. However, this prediction did not hold. Instead, no change in spatial gain between the pre-disparity and post-disparity blocks is observed (model II SMA regression line intercept = -0.06, slope = 1.04, confidence intervals include the unity slope line). Therefore, auditory shift appears to be insensitive to local patterns of encoded disparity across space. Taken together, these results indicate that only bias is changing in response to repeated exposure to a fixed AV disparity, and not gain.

## Experimental discussion

We measured shifts in auditory spatial perception following AV disparity trains to investigate the time course of adaptive changes in auditory spatial perception. We found evidence for both brief, transient shifts in auditory perception following each AV disparity train, as well as slower, more enduring shifts over the course of each session. Our results parallel recent findings in AV *temporal* disparity [30], suggesting that audio-visual calibration is generally maintained over multiple time scales. The difference in auditory shift in the “same location” and “different location” in this experiment demonstrated that, following brief exposure to spatially disparate AV targets, auditory spatial perception undergoes transient shifts. Because we varied both delay and distance of different location targets from the preceding AV disparity these data do not allow us to directly compare the effects of time and space. However, our previous work [16] has shown that both delay and distance from the AV disparity significantly reduce auditory shift, so we expect that both are at play in this experiment as well. We note that the “different location” targets had auditory shift that was elevated above a line connecting the pre-disparity to post-disparity auditory shifts, suggesting that the transient component of the VAE influenced localization of these targets. We found this surprising, because our previous work suggested that the VAE should have almost fully dissipated in these trials. This discrepancy could be because in our previous experiment we alternated the direction of AV disparity between AV disparity trains, which would prevent the VAE from accumulating in the same direction across trials. Alternately, the decay of auditory shift may change when the AV disparity has a consistent direction across presentations. We further explore the temporal and spatial mechanisms that could explain the observed data in the following modeling section. Additionally, the difference in auditory shift between the pre-disparity and post-disparity blocks in this experiment demonstrated that prolonged exposure to constant AV disparity produces a gradual shift in auditory spatial perception. This observed recalibration of auditory spatial perception after exposure to a fixed AV disparity is consistent with previous studies [7, 8, 18, 19]. The observed shift in auditory spatial perception affected auditory perception over the entire target range and did not diminish over the measured time period. These results suggest a slower, enduring recalibration of auditory spatial perception.

In our analyses, we assumed that subjects only have access to the *encoded* spatial disparity in AV targets. Therefore, initial offset should influence recalibration by summing with physical disparity to produce encoded disparity (Eq 1). Our results demonstrate that recalibration is proportional to initial mean encoded disparity (Fig 4, left panel). This result agrees with previous results demonstrating that recalibration also scales with *physical* AV disparity [8], because encoded disparity is dependent on physical disparity as well as auditory offset. However, future studies should measure or control for encoded offset in auditory spatial perception on a subject-by-subject basis. Despite the dependence of recalibration on the encoded offset between targets, auditory spatial gain did not change in response to AV disparity. This result suggests that recalibration is based on average AV disparity, *not* local AV disparity at different places in space (Fig 4, right panel). However, previous experiments that specifically manipulated spatial gain with minifying lenses that compressed visual space to 0.5x the normal visual field showed that, when subjects wore these lenses over the course of days, partial recalibration of auditory spatial gain could be elicited [9]. Therefore, the negative result we obtained could be because our experiments were not long enough to observe similar recalibration of spatial gain, or because we did not specifically alter the AV disparity across space. Within the context of the current experiment, encoded AV disparity is averaged across space and applied as a uniform recalibration of auditory spatial perception. Due to the relatively short duration of the current experiment, no subject shifted to the point at which the mean encoded AV disparity was zero. We predict that a longer experiment, in which the mean encoded difference in auditory and visual target location reached zero but patterns of error were still present across space (due to non-unity spatial gain), would reveal changes in spatial gain as well.

These results demonstrate how the VAE acts over multiple time scales, but cannot determine the underlying mechanisms without a direct comparison of the predictions those mechanisms make about the data. In the following section, we propose candidate models to explain these data, fit them to data from each experimental session, and compare goodness of fit to determine the best explanation for these results. Because our experimental data demonstrate that a good model must be able to produce both large, transient shifts as well as slow, enduring shifts, we focus on models that can span multiple time scales. Additionally, we test these temporal models both with and without a spatially focal component, to determine if these data support the idea that the VAE dissipates with distance from the location of an AV disparity.

## Modeling methods

**Fig 5:**
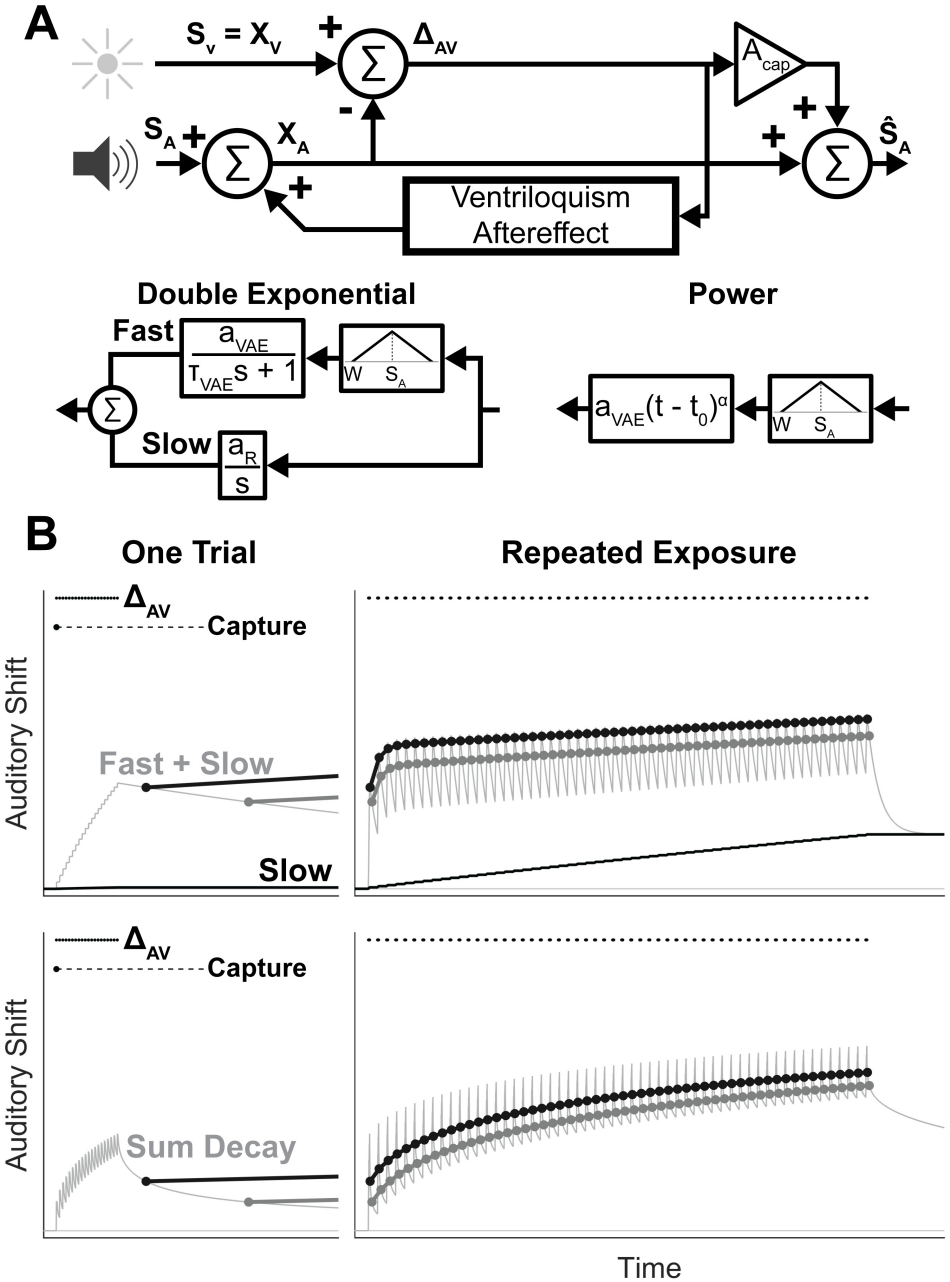
Model of the ventriloquism aftereffect. **A**, Block diagram summarizing the state-space model, along with the double exponential and power models of the ventriloquism aftereffect. **B**, Example model responses to AV disparity trains. The top panels show the double exponential model, and the bottom panels show the power model. The left panels show how auditory shift builds and decays to a single AV disparity train (each dot represents a single repetition of the AV disparity). When auditory targets are presented following an AV disparity train, auditory shift influences the encoded location of the target (points and trend lines, colored to match Fig 3). Additionally, visual capture (not measured in the present experiment) occurs within the first AV disparity and can be held in memory indefinitely, as shown in [16]. The right panels show how both models can replicate the trends in auditory shift produced by repeated exposure to a fixed AV disparity observed in Fig 3. In both panels, median best fit model parameters across participants were used to produce the trends shown, to allow for visual comparison of model predictions. The effect of changing spatial location was not shown here to emphasize the changes in temporal relationship across the two models, so the difference in auditory shift across trial types appears smaller than in Fig 3.

This model describes the influence of vision on auditory spatial perception demonstrated in the previous experiment. We model the general structure of these phenomena as a state-space system that processes a sequence of auditory and audio-visual targets to estimate encoded auditory target location, as summarized in Fig 5A. Based on the experimental data, we model three alternative temporal accumulation and decay processes, each with or without a spatial decay component. In the double exponential model, auditory shift is implemented by an exponentially accumulating and decaying fast process superimposed on a slow, persistent integrator. A single exponential version of this model was also tested, in which the slow integrator was absent. Finally, in the power model, the temporal decay of the ventriloquism aftereffect was modeled with a power relationship, which provides a long tail that can build up over time. Model free parameters were estimated with nonlinear gray-box model estimation (nlgreyest) in the system identification toolbox of MATLAB, and our implementation and data are available in S1 File.

### Initial states

We assume that subjects start with a calibrated map of auditory space that is unique to each individual. Previous experiments have demonstrated that subjects tend to have small, uniform biases in auditory spatial perception (denoted *μ*_*A*_), and a tendency to overestimate auditory target azimuth (also referred to as spatial gain, *SG*_*A*_), as described by Eq 1. The current calibration of auditory space is denoted *R*_*z*,*k*_, which is the shift in auditory spatial perception at azimuth, *z*, for the *k*th target in the experiment. Mean encoded auditory target location at the start of an experiment can be calculated by adding *R*_*z*,0_ to target location, and is given as:

$$\begin{array}{c} R_{z,0}=z*\left(SG_{A}-1\right)+\mu_{A} \end{array}$$ (2)

Experimentally, we obtain an estimate of *R*_*z*,0_ by having subjects localize a sequence of auditory targets over a range of azimuths, then performing a first-order linear fit to the pointing responses as a function of target location, as shown in Fig 2. The slope of this fit gives *SG*_*A*_, and the intercept gives *μ*_*A*_. Perfect accuracy occurs when *SG*_*A*_ = 1 and *μ*_*A*_ = 0, which would lead to *R*_*z*,0_ = 0 at all azimuths. Overshoot and undershoot occur when *SG*_*A*_ > 1 or *SG*_*A*_ < 1, respectively. Uniform biases toward the left or right occur when *μ*_*A*_ < 0 or *μ*_*A*_ > 0, respectively.

The transient component of the ventriloquism aftereffect is denoted *VAE*_*z*,*k*_, which is the effect of this process on auditory perception at azimuth *z* for the *k*th target in the experiment. At the start of an experiment subjects have not viewed any audio-visual target pairs, so we assume that its effect is zero at all azimuths:

$$\begin{array}{c} VAE_{z,0}=0,\forall z \end{array}$$ (3)

### Inputs

Auditory and visual targets are presented from independent locations, *S*_*A*,*k*_ and *S*_*V*,*k*_, respectively. These target locations are encoded by the nervous system, as represented by *X*_*A*,*k*_ and *X*_*V*,*k*_. Auditory spatial perception is influenced both by recalibration and visual capture, so encoded auditory target location is given as the true target location plus the ventriloquism aftereffect:

$$\begin{array}{c} X_{A,k}=S_{A,k}+R_{S_{A,k},k}+VAE_{S_{A,k},k} \end{array}$$ (4)

For the sake of simplicity, we assume that visual errors are negligible, that vision does not shift in response to AV disparities, and that the encoded visual target location is the true target location:

$$\begin{array}{c} X_{V,k}=S_{V,k} \end{array}$$ (5)

Note that *X*_*V*,*k*_ does not exist if no visual target was presented. Additionally, this model does not include sensory uncertainty, which adds normally-distributed noise to encoded target location.

Experimentally, we introduce spatial disparities between auditory and visual target locations to elicit changes in auditory spatial perception. Here, we assume that subjects only have access to the distance between encoded auditory and visual targets, and not the true distance between them. When audio-visual target pairs are presented, we calculate the difference between their encoded locations as Δ_*AV*,*k*_:

$$\begin{array}{c} \Delta_{AV,k}=\{\begin{array}{cc} X_{V,k}-X_{A,k}, & \text{if}\;X_{V,k}\;\text{exists}\\ 0 & \text{otherwise} \end{array} \end{array}$$ (6)

### State equations

A spatial disparity between auditory and visual targets causes the ventriloquism aftereffect to occur. In all three models, AV disparities elicit the transient component of the ventriloquism aftereffect, *VAE*_*z*,*k*+1_, which is a function of the magnitude of the encoded AV disparity and time since the preceding AV disparities. In both exponential models, *VAE*_*z*,*k*+1_ is defined as:

$$\begin{array}{c} VAE_{z,k+1}=\left(VAE_{z,k}+a_{VAE}*\Delta_{AV,k}*\text{SpatialWindow}\right)*\text{Decay} \end{array}$$ (7)

*a*_*VAE*_ determines the rate of increase of the auditory shift. The exponential and power models differ in how the define the temporal decay. For the exponential model, decay is defined as:

$$\begin{array}{c} {\text{Decay}}_{\text{Exponential}}=e^{\frac{-ISI_{k,k+1}}{\tau_{VAE}}} \end{array}$$ (8)

Decay_Exponential_ models the forgetting process as a first-order exponential decay with time constant *τ*_*VAE*_. *ISI*_*k*,*k*+1_ is the time between the current and the next target in the sequence, measured from their respective onsets.

Because power law relationships are not stateless, for the power model, *VAE*_*z*,*k*+1_ is defined independently for each preceding disparity and the net effect of VAE at each time point is the sum of each of these decay time courses.

$$\begin{array}{c} {\text{Decay}}_{\text{Power}}={\left(t-t_{0}\right)}^{-\alpha }*{\left(0.05\right)}^{\alpha } \end{array}$$ (9)

In Decay_Power_, *t* is the current time, *t*_0_ is the onset time of the AV disparity, and *α* is the rate of decay. Because each target was 50 ms long, the (0.05)^*α*^ term was included to normalize the decay function so it starts with a value of 1 at 50 ms.

The definition of *VAE*_*z*,*k*+1_ also includes a spatial window, to represent the diminishing effect of the ventriloquism aftereffect with distance from the AV disparity location. We modeled the spatial window as a symmetric triangle centered on the auditory target location with a peak of 1 and a width of *W* (i.e. the distance from the auditory target where the triangle crosses zero):

$$\begin{array}{c} \text{SpatialWindow}=max(0,1-\frac{abs(z-S_{A,k})}{W}) \end{array}$$ (10)

All three temporal models were tested with and without the spatial window, to determine if it was necessary to explain the data. The spatial window was removed by setting it to 1 across all azimuths.

In the double exponential model, persistent changes in auditory spatial perception were modeled as changes to calibration. If target *k* contains an AV disparity, Δ_*AV*,*k*_, it elicits a change in subsequent auditory perception, *R*_*z*,*k*+1_, which is modeled as:

$$\begin{array}{c} R_{z,k+1}=R_{z,k}+a_{R}*\Delta_{AV,k} \end{array}$$ (11)

*a*_*R*_ governs the rate at which recalibration occurs. In the single exponential model, *a*_*R*_ was set to zero. Here, we do not model location-specific changes in recalibration, but this could be achieved by modifying the calculation of *R*_*z*,*k*+1_ to vary as a function of azimuth, *z*.

The double exponential model handles the persistent auditory shift in the post-disparity block directly through recalibration of *R*_*z*_, whereas it is explained in the power law model by the sum of the long tail of previous exposures in *VAE*_*z*_. The power model predicts that auditory shift should gradually decay in the post-disparity block, although our post-disparity block was not long enough to determine whether or not this prediction holds.

### Output

This model predicts subject responses when localizing the auditory component of target *k*, denoted ${\hat{S}}_{A,k}$. Although we did not measure it in this experiment, we included visual capture in the model to facilitate the model’s use in future experiments. For simplicity, we assume that visual capture always occurs at a fixed proportion of Δ_*AV*,*k*_, denoted *A*_*Cap*_, rather than modeling the chance that visual capture does or does not occur on a trial-by-trial basis [19, 27, 31]. Experimentally, the distance between targets is kept relatively small(*S*_*A*_ − *S*_*V*_ = ±8° for all experiments), so capture should occur on most, if not all, audio-visual targets. If the target had no visual component, the response is simply the encoded auditory target location *S*_*A*,*k*_.

$$\begin{array}{c} {\hat{S}}_{A,k}=\{\begin{array}{cc} X_{A,k}+A_{Cap}*\Delta_{AV,k}, & \text{if}\;X_{V,k}\;\text{exists}\\ X_{A,k} & \text{otherwise} \end{array} \end{array}$$ (12)

## Modeling results

Comparisons of the single exponential, double exponential, and power models, both with and without spatial windows, are summarized in Table 1. Best fits were obtained for each experimental session with each model using nonlinear gray-box model estimation, and the goodness of fit for each model for each session and across all sessions were compared to identify the models that could best explain these data. Normalized root mean squared error (NRMSE) was calculated to assess how well the models fit the data, and was defined as the root mean squared error of the model fit divided by root mean square difference between each data point and the mean of the data. A NRMSE value of 1 indicates perfect fit, while a value of 0 indicates that the model is no better than the mean of the data. All tested models explained the data much better than an intercept only model, with NRMSE values greater than 0.99 for all models across experimental sessions. Because this metric of fit quality produced only small differences between models, we instead used Akaike’s Information Criteria [32], corrected for sample size (AICc), to compare model fits for each experimental session. Both the power and double exponential models with spatial windows performed well, with each model providing the best fit for 12 of the 24 experimental sessions. Median AICc was 4291 and 4330 for the power with spatial window and double exponential with spatial window models, respectively. The median and sum difference in AICc across these models slightly favor the power law model, but not substantially. Therefore, it seems that both models are equally good representations of the experimental data, and that additional experimentation would be necessary to identify which is correct. As noted in [33], power-law adaptation can arise from the sum or cascade of multiple exponential functions with different time constants, suggesting that the similarity in model fit quality may arise from the similarity of these two approaches to modeling temporal dynamics. Examples of both model fits, using the median parameters across all experimental sessions, is provided in Fig 5B. As shown, both models produce a gradual growth of auditory shift, along with a jagged time course that alters the measured auditory shift depending on the time time delay between the AV disparity and subsequent auditory targets. Both models can reproduce the trends observed in the group results shown in Fig 3.

**Table 1:** Model fit comparison. Median and sum differences in Akaike’s Corrected Information Criteria (AICc) between each model and the best fitting model (power with spatial window) are reported, sorted by median difference.

Model	Median Δ AICc	Sum Δ AICc
Power With Spatial Window	0	0
Two Exponential With Spatial Window	2.5	66
Power	5.8	2170
Two Exponential	16.2	1603
Single Exponential With Spatial Window	33.1	1650
Single Exponential	84.9	2974

The rest of the models performed worse than the top two. Qualitative comparison of the differences suggests that the utility of the spatial window varied across individuals, with the model fitting some subject data just as well for some, but substantially worse for others, when the spatial window was not used. The single exponential model performed much worse than the double exponential and power models, because it could not account for the sustained auditory shift in the post-disparity block. The single exponential model fits attempted to compensate by increasing the decay rate *τ*_*VAE*_, but this was insufficient to match the performance of the other models.

Parameter estimates are summarized in Table 2. Across both models, the spatial window size was very similar, but the rate of increase of the auditory shift, *a*_*VAE*_, differed by about an order of magnitude. This is evident in the simulations shown in Fig 5B, which show a staircase pattern for the response to one trial in the double exponential model, but a jagged rising edge for the power model. Apparently, the power model requires more accumulation of shift to compensate for the rapid initial drop afterward, whereas the slower exponential time course does not require as steep of a drop. The time constants of the two models are not directly comparable, but both model fits are capable of producing the observed trends. Across individuals, parameter values spanned a wide range around the group median, so the model fit parameters given here should be interpreted as rough estimates intended to guide future experimental design, rather than definitive quantification of the ventriloquism aftereffect.

**Table 2:** Model best fit parameters. Values indicate population median, and were used to produce the model simulations in Fig 5B.

Power With Spatial Window	*a*_*VAE*_	*W*	*α*	
	0.26	29.5°	0.51	
Double Exponential With Spatial Window	*a*_*VAE*_	*W*	*τ*_*VAE*_	*a*_*R*_
	0.02	28°	51 s	0.0003

## Modeling discussion

Both the double exponential and power models can produce the temporal dynamics of the ventriloquism aftereffect, indicating that the ventriloquism aftereffect is best described as having a rapid, transient component that gives way to a slower, more enduring component. This finding is in agreement with the previous experimental literature. *a*_*VAE*_ is sufficient to produce shifts in auditory perception following a single train of AV disparities in both models, as shown in the one trial examples in Fig 5B. The staircase pattern of the double exponential model seems less plausible given the fact that previous studies have demonstrated significant auditory shifts following a single audio-visual target [14, 16]. However, the number of AV disparities in each train was not varied in this experiment, so it is possible that both models would reach solutions that could produce a significant shift after a single AV disparity if trained with data from these studies. The spatial window significantly improved the quality of the model fit, which supports previous findings that the ventriloquism aftereffect is spatially focal around the location of the AV disparity [10, 16, 17]. Additionally, the value of *W* in both models appears to be in agreement with previous studies that provided audio-visual disparities at a constant location in space [17]. In both models, the rate at which the long-term effects of the ventrioquism aftereffect build up is slow. This predicts that extensive experience is necessary to produce a lasting recalibration of auditory perception, in agreement with previous studies [7, 8, 18, 19]. Each model makes slightly different predictions about the time course of the slow components of the ventriloquism aftereffect. The power model predicts that we should have seen more curvature in the pattern of auditory shift early in the exposure to disparity block, but such curvature is not evident in the group trend in Fig 3. Additionally, both models predict that the beginning of the post-exposure block should have a temporally decaying component. We found that auditory shift in the post-disparity block was not significantly different across time, but visual inspection suggests that there might be a non-significant decay in auditory shift over the first few trials of the post-disparity block. The power model predicts that auditory shift should continue to decay over time, whereas the double exponential model predicts that it will persist indefinitely. It seems unlikely that auditory shift would remain indefinitely following the end of exposure to AV disparity, but our post-disparity block was not long enough to test this prediction.

Although we currently don’t have data to further refine this model, we predict that some of the components are likely a simplification of the true processes that occur in response to audio-visual spatial disparities. First, the equivalence of the dual exponential and power models may simply reflect the design of the experiment, and alternate experimental designs will be necessary to determine the true structure of the temporal dynamics of the ventriloquism aftereffect. Specifically, we did not design to measure the accumulation of shift in each AV disparity train, the decay of shift following the end of exposure, or the trend at the onset of the exposure to AV disparity block, which could all be of use in future experiments to determine the temporal dynamics underlying the ventriloquism aftereffect. Second, we randomly varied the spatial relationship between AV disparities and subsequent auditory targets, but not in a manner that would allow us to make strong claims about the shape of the spatial window of the ventriloquism aftereffect. A triangle was selected because it appeared to qualitatively follow the trend in previous studies, but other functions could produce similar results. Third, we observed a substantial variability in most model parameters across subjects, suggesting that a more detailed analysis of individual variability is necessary to determine whether these models can be assumed to generalize across individuals. Fourth, it is possible that manipulations of experiment design could alter some or all of the measured model parameters, which would need to be tested across similar experiments within the same subjects.

This model predicts that the rapid, transient components of the ventriloquism aftereffect should decrease the rate of recalibration when audio-visual target pairs are presented in bursts. This decrease in recalibration is predicted to occur because the rapid buildup of auditory shift would decrease the mean encoded audio-visual disparity when audio-visual target pairs are presented in rapid succession, as in the AV disparity trains in the current experiment. A future experiment could test this prediction by presenting the same number of AV target pairs, but with different temporal grouping.

## Conclusion

The experimental data presented here demonstrates how the ventriloquism aftereffect occurs over different time scales, and how repeated exposure to brief AV disparities alters auditory perception by producing both a large, transient initial shift, as well as a smaller, more enduring shift. Two different models were equivalent in their ability to explain the measured temporal dynamics. The power model’s temporal decay of shift was initially rapid, but persisted for a long time, so that toward the end of the experiment auditory spatial perception was shifted by the accumulation of these decay curves. In contrast, the double exponential model’s transient shifts completely disappeared after a fixed period of time, and the enduring shift at the end of the experiment was produced by a second, slow accumulator. Additional work is needed to determine which model best explains the underlying mechanisms of the ventriloquism aftereffect, but overall, our findings demonstrate the importance of considering how the ventriloquism aftereffect evolves over multiple time scales.

## Supporting information

S1 File Experimental data and computational models. Data are provided as .csv files, and can be readily parsed by BuildRecalibrationInputVector.m. All analyses were conducted with MATLAB 2017b. Computational modeling requires the System Identification Toolbox, and can be run by calling EstimateModelParameters.m.
(ZIP)
